# Super-enhancers in transcriptional regulation and genome organization

**DOI:** 10.1093/nar/gkz1038

**Published:** 2019-11-14

**Authors:** Xi Wang, Murray J Cairns, Jian Yan

**Affiliations:** 1 Key Laboratory of Resource Biology and Biotechnology in Western China (Northwest University), Ministry of Education / School of Life Sciences, Northwest University, Xi’an 710069, China; 2 Division of Theoretical Systems Biology, Germany Cancer Research Center, Heidelberg 69115, Germany; 3 School of Biomedical Sciences and Pharmacy, Faculty of Health and Medicine, The University of Newcastle, University Drive, Callaghan, NSW 2308, Australia; 4 Centre for Brain and Mental Health Research, University of Newcastle, Callaghan, NSW 2308, Australia; and Hunter Medical Research Institute; 5 Department of Biomedical Sciences, City University of Hong Kong, Kowloon Tong, Hong Kong S.A.R., China

## Abstract

Gene expression is precisely controlled in a stage and cell-type-specific manner, largely through the interaction between *cis*-regulatory elements and their associated *trans*-acting factors. Where these components aggregate in promoters and enhancers, they are able to cooperate to modulate chromatin structure and support the engagement in long-range 3D superstructures that shape the dynamics of a cell's genomic architecture. Recently, the term ‘super-enhancer’ has been introduced to describe a hyper-active regulatory domain comprising a complex array of sequence elements that work together to control the key gene networks involved in cell identity. Here, we survey the unique characteristics of super-enhancers compared to other enhancer types and summarize the recent advances in our understanding of their biological role in gene regulation. In particular, we discuss their capacity to attract the formation of phase-separated condensates, and capacity to generate three-dimensional genome structures that precisely activate their target genes. We also propose a multi-stage transition model to explain the evolutionary pressure driving the development of super-enhancers in complex organisms, and highlight the potential for involvement in tumorigenesis. Finally, we discuss more broadly the role of super-enhancers in human health disorders and related potential in therapeutic interventions.

## INTRODUCTION

Enhancers are well known for their genomic location and orientation independent activity in the regulation of gene expression. The first eukaryotic enhancer was identified in a primate virus SV40 in the early 1980s. Banerji *et al.* observed that the remote viral element containing a 72-bp repeat sequence could enhance recombinant β-globin expression 200-fold when cohabitating in a plasmid construct transfected into mammalian cells (1). Two years later, several independent groups reported that enhancers located in the mouse immunoglobin (*Ig*) heavy chain gene loci could activate nearby *Ig* promoter in *cis* specifically in lymphocytes, which demonstrated for the first time that enhancers function in a tissue specific manner (2–4).

The term ‘super-enhancer’ was first used in 2004 by Chen and colleagues to describe a 651 bp segment of baculoviral genomic DNA designated *hr3*. They observed that this regulatory domain could stimulate activity of the *ie-1* reporter gene promoter up to 7000-fold in transfected cells (5). Almost a decade later, Young and colleagues used the term ‘super-enhancer’ (SE) to characterize large genomic domains, conferring a key role in control of cell identity and disease (6–8). Using ChIP-seq data from the multiple tissue types available from the ENCODE and Roadmap Epigenome Projects (9,10), they were able to demonstrate that SEs span tens of kilobases (kb) of DNA sequence and are densely occupied by master transcription factors (TFs) and mediators. Collectively, these observations suggested that SEs play a key role in organizing the gene expression patterns that regulate cell identity (6–8). The Young definition of SE, in relation to developmentally important genomic segments, extends well beyond the early usage, which related to their performance in expression assays *in vitro*, and has become the established use of the terminology.

While there is now very substantial support for a paradigm in which SEs are a major regulatory component of the gene expression that shapes cell identity, there is an alternative view held by some researchers, that SEs are no more than clusters of enhancers (11,12) that contribute with additive effect on their target genes in a manner more similar to previously described locus control regions (LCRs) (13). Therefore, in view of this controversy, we consider it is timely to review our current knowledge of SEs and discuss the evidence in support of the range of opinions. In this work, we also recapitulate genome-wide identification and characterization of SEs and provide an online repository of a high-quality collection of SEs with meta-analysis. Furthermore, we explore the biological support for a role of SEs in gene regulation in light of the phase separation and three-dimensional (3D) genome organization models for SE action. We also propose an evolutionary framework to explain the emergence of SEs in complex organisms. Finally, we will discuss the involvement of SEs in human health disorders and their potential as targets for therapeutic interventions.

## GENOME-WIDE IDENTIFICATION OF SUPER-ENHANCERS

**Figure 1.**
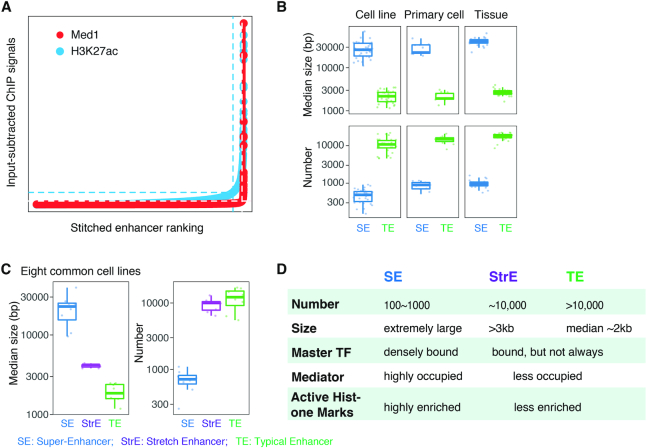
Identification and characteristics of super-enhancers. (**A**) Call of SEs with MED1 or H3K27ac ChIP-seq data, using ROSE algorithm which takes into account enhancer ranks and ChIP signals (6). Y-axis gives input-subtracted MED1 or H3K27ac ChIP-seq coverage, and x-axis shows the rank of ‘superness’ based on the value given on y-axis. Dashed lines in both directions indicate the cutoffs for separating SEs from typical enhancer. (**B**) Box-plots comparing the median size (upper) and the number (bottom) between SEs and typical enhancers (TEs) in 30 cell lines, 11 primary cells and 24 tissues available from the ENCODE project. (**C**) Box-plots comparing the median size (left) and the number (right) of SEs, stretch enhancers (StrEs), and typical enhancers (TEs) in eight selected cell lines. (**D**) Characteristic summary of SEs, in comparison to stretch enhancers (StrEs) and typical enhancers (TEs).

Young and colleagues originally identified SEs at the genome-wide scale, based on ChIP-seq signal enrichment of Mediator subunit MED1 or master TFs, such as MyoD, T-bet and C/EBPα, in mouse embryonic stem cells (mESCs) and other tissues including myotube, T helper and macrophages (6,7). A similar strategy was also applied to other cell types using ChIP-seq data of acetylated histone H3 lysine 27 (H3K27ac), which is a surrogate epigenomic marker of active enhancers (8). The Young group also developed the ROSE software tool to facilitate SE identification *in silico* (7,8). This algorithm stitches closely-distributed enhancers identified from H3K27ac (or MED1/master TF) ChIP-seq data, ranks the stitched enhancers by their input-subtracted ChIP-seq signal, and finally separates SEs from typical enhancers by a graphic elbow point identified on the ranked ChIP-seq signal plot (Figure 1A). The output is slightly different for the different kinds of data input, such that the elbow points are usually sharper with MED1 than H3K27ac, and the final SE collections identified by the two marks are not in 100% agreement. To exclude the possibility of transcription start sites (TSS) overlapping with regions of SE calling, constituent enhancers are usually excluded from stitching if they are located within a ±2000 bp window flanking an annotated TSS (8).

To the best of our knowledge there are currently three SE databases which gather published SEs and implement the ROSE algorithm to mine available ChIP-seq data, including dbSUPER (14), SEA (15), and SEdb (16). The most recent of these, SEdb, contains a collection of more than 331,000 SEs derived from 541 human cell lines/tissues. We also provide an online data repository of SE data, including a core collection of human SEs with comparative and exploratory analyses (discussed below in this Survey and Summary) to further support the biological investigation of these structures. This resource is available at https://sunlightwang.github.io/Super-Enhancers/ and will be continuously updated and expanded going forward.

## UNIQUE CHARACTERISTICS OF SUPER-ENHANCERS

### SEs are comprised of a small number of genomic loci of extremely large size

In a comparison of SEs and typical enhancers (TEs) in 30 cell lines, 24 tissues and 11 primary cell types available from the ENCODE project (10), it was noted that the median size of SEs in general spreads from 10 kb to over 60 kb, whereas the median size of TEs ranges from 1 kb to 4 kb, smaller by approximately one order of magnitude (6,7) (Figure 1B, upper). By contrast, when looking at the number of SEs and TEs in each cell type, the trend is exactly the opposite: SEs are fewer than TEs by one to two orders of magnitude (Figure 1B, bottom).

Around the time that these SE were described, another group independently reported enhancers of size >3 kb, and used the alternative nomenclature ‘stretch enhancer’ (StrE) to characterize their extraordinary length (17). Similar to SEs, StrEs are also found cell type specific and important in programming cell identity gene expression (17). Although SEs and StrEs share some properties, they are conceptually and functionally different in at least two respects. Firstly, while StrEs are determined by an arbitrary cut-off in genomic size (3 kb), SEs are discriminated from other enhancers in a parameter-free manner after clustered enhancers stitching (Figure 1A), which gives more weight to the biological essence of SEs. Secondly, extraordinarily strong TF binding and associated Mediator complex signals endow SEs with special biochemical properties, for example, facilitating them to form liquid-liquid phase-separated condensates (discussed later in this article). By contrast, any large enhancer can be designated a StrE, regardless of their biological activity.

In a comparison of SEs with TEs, StrEs in eight cell lines where collections of all the three enhancer types are available, both the small-number and extremely-large-size of SEs become more apparent (Figure 1C). For example, the median size of StrEs is in the order of a thousand base pairs, 2- to 3-fold larger than that of TEs, but still much smaller than their SE counterpart. While SEs are usually stitched up by constituent enhancers, between which there can be gaps of up to 12.5 kb (a cut-off used in the original SE paper), StrEs are defined as large enhancers or enhancer-like chromatin states based on hidden Markov model inference. Therefore, some caution should be applied where large gaps may prevent an account of synergistic influences from nearby domains in a StrE. When looking at the number of the three types of enhancers in each cell line, the StrEs are numerous, much more comparable to TEs than SEs (Figure 1C, D).

### SEs specify cell identity

**Figure 2.**
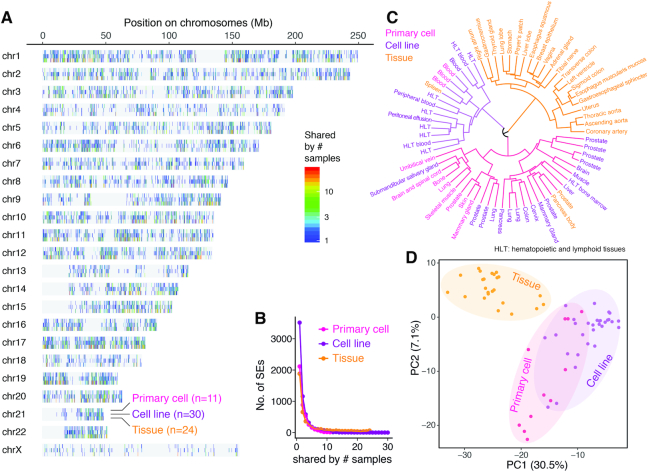
Super-enhancers and cell identity. (**A**) Genome-wide SE landscape of in total 65 samples (30 cell lines, 11 primary cells and 24 tissues) available from the ENCODE project. Bars on the chromosomes indicate the genomic loci of SEs, and colors indicate each SE appearing in how many different cell lines, primary cells, and tissues, respectively. (**B**) The number of SEs (y-axis) co-occurring in different number (x-axis) of cell lines, primary cells and tissues, respectively. The vast majority of SEs are not shared by different samples. (**C**) Hierarchical clustering of the 65 samples based on co-occurrence of SEs measured by the pairwise Jaccard distance. The colors in the dendrogram indicate the three major clusters, and the text colors of the sample names indicate the sample types (i.e. cell lines, primary cells or tissues). (**D**) Principal component analysis (PCA) of the 65 samples based on the SE co-occurrence matrix. The scatter plot shows the first two PCs, and the dot colors indicate the sample types (i.e. cell lines, primary cells, or tissues).

Within the high-quality SE collections derived from the ENCODE project, including the data from 65 samples described above, we charted the genome-wide SE landscape as shown in Figure 2A. The vast majority of SEs only appear in few or individual cell types (Figure 2B), supporting the assertion that SEs are highly cell type specific. To further explore this specificity, we performed hierarchical clustering based on the Jaccard distance that measures the SE location dissimilarity between every pair of all the 65 samples. A larger Jaccard distance is indicative of fewer overlapped SEs relative to the total number of all SEs occurring in the two sample comparison, and vice versa. As shown in Figure 2C, the hierarchical clustering indicates a clear separation between tissue samples (except for only three outliers) and cell lines / primary cells, indicating that SEs are also sensitive to the cellular growth environment. Indeed, environment-specific SEs were observed in resident macrophages (18). Principal component analysis (PCA) based on SE occurrence matrix of the 65 samples also confirmed the same phenomenon, even with a much clearer segregation of tissue samples from the others (Figure 2D). Primary cells scatter between tissue samples and cell lines when looking at the first component (i.e. the x-axis of Figure 2D), which accounts for nearly one third of the variance, confirming that both the growth potential and immortality of primary culture cells lie in between the tissue samples and cell lines. Intriguingly, the PCA visualization of the 65 samples based on SE occurrence is analogous to the analysis based on global gene expression, where cell lines show distinct gene expression profiles compared to normal tissues (19). It is also interesting to note that sub-clusters comprised of only primary cells or cell lines are observed, despite the fact that they are, in general, more intermingled (Figure 2C, D). When looking at a finer resolution in Figure 2C, cell lines derived from similar cell origins (e.g. prostate or blood), tend to be grouped together immediately, which is consistent with literature reports (8). Such phenomenon is also observed in tissues developed from a common pathway (e.g. thoracic aorta, ascending aorta and coronary artery).

These observations suggest that SE profile can be used as a biomarker or proxy to categorize cell types and their cell growth conditions or status. Moreover, the fact that SEs differ among the same cell type in different niches also suggests that they are sensitive to external environmental signals (18). To date, SEs are becoming commonly used as molecular markers to sub-classify complex diseases in precision medicine, and this will be discussed later in this article.

### SEs are densely bound by master TFs

It has been shown that enhancers can interact with promoters via long-range chromatin loops to activate gene transcription (20). These structures are mediated and stabilized by the complex cooperation between an array of *cis*-acting regulatory elements and *trans*-acting proteins that co-localize in each chromatin domain. In both human and eukaryotic model organisms, TFs are found densely bound to enhancers in a combinatorial manner and synergistically responsible for regulatory activity (21–26). SEs are densely co-occupied by master TFs and Mediator complex, and these master TFs establish auto-regulatory networks (6,27,28). In mouse ESCs, the three well-known pluripotent TFs, Oct4, Sox2 and Nanog are identified at SEs at extraordinarily elevated levels (6). In comparison to StrEs, SEs have a significantly higher level of chromatin accessibility surveyed by DNase I hypersensitivity assay and also denser master TF binding signals (29).

Interestingly, TF binding sites inside SEs are not evenly distributed but form a series of dense clusters, termed ‘constituents’ (6), ‘epicenters’ (30) or ‘hotspots’ (27). In a more dynamic system, Adam and colleagues took advantage of the hair follicle stem cell differentiation model, and observed the remodeling of SEs and their epicenters during lineage commitment (30). The cohort of TF binding sites was also altered following the change of SE locations. The location alteration was reversible and remarkably sensitive to microenvironment, allowing high plasticity for cells to adapt the environment for proliferation and lineage commitment. In this system, the pioneer factor SOX9 took the major responsibility to govern the landscape of SEs by protecting against H3K27me3 (histone H3 lysine 27 tri-methylation, a marker for inactive chromatin) and initiating enrichment of H3K27ac. In the aforementioned example, macrophages residing in five different tissue environments share only ∼40–50% of SEs (18). The pioneer factor PU.1 is a fate-determining TF for transmitting the environmental signal to commitment of environment-specific SEs in macrophages.

Several lines of evidence suggest that master TFs are capable of directing active SE formation (18,30,31) and this is supported by extensive co-localization of TFs within SE ‘hotspot’ regions either sequentially or simultaneously in response to developmental stimuli (27). Hnisz *et al.* demonstrated that terminal TFs of multiple signaling pathways frequently co-occurred in SEs but not their smaller TEs cousins, suggesting that SEs may provide a ‘hub’ for cells to be hyper-sensitive to divergent environmental signals (32).

### High occupancy of mediator at SEs

Defined by the enhancer clusters with high Mediator occupancy and/or strong enhancer activity, SEs are expected to largely overlap with known functional chromatin domains, such as differential methylation regions (DMRs), locus control regions (LCRs) and transcription initiation platforms (TIPs) (8). Compared to stretch enhancers and typical enhancers, SEs exhibit significantly higher enrichment of active chromatin marks and binding of chromatin remodelers and organizers (29). The Mediator complex, composed of 26 core subunits, is known to be associated with enhancers and mediate enhancer functions by transmitting regulatory signal to the associated transcription machinery (33–36). Mediator contributes to stable assembly of transcription pre-initiation complex, regulation of RNA Polymerase II (PolII) pausing and elongation, and the formation of enhancer-promoter looping and three-dimensional (3D) genome organization (reviewed in (34)). SEs are heavily loaded with the Mediator complex at least one order of magnitude greater than any typical enhancers (6), giving rise to extraordinary activity and specific biochemical characteristics (7).

Altogether, SEs demonstrate their ‘superness’ not only by their enormous size, achieved though clustering closely localized enhancers, but also their super-strong transcriptional activity due to dense interaction with transcription factors, chromatin remodelers, transcription co-activators, and Pol II holoenzyme (Figure 1D). These properties are further supported by their capacity to drive short and long range interaction through phase separation and 3D genomic association, highlighting the qualitative difference to typical enhancers (see below), and suggest a mechanism to explain why transcriptional activation by SEs is greater than the sum of their constituent enhancer components (12).

## THE ROLE OF SUPER-ENHANCERS IN TRANSCRIPTIONAL REGULATION

Given the unique characteristics of SEs, it is interesting to speculate why complex cells need them? The most likely answer to this question is that SEs specify and maintain cell identity, which is a vital biological attribute of complex multicellular organisms that need to be able to developmentally regulate the formation and maintenance of cell type specific tissue compartment. While mechanisms for transcriptional regulation through promoter activation were first identified in unicellular prokaryotes (37), the level of sophistication in vertebrate systems probably required more mechanistic complexity including long-range chromatin interactions from enhancers to target genes (20). Cell type specificity, in particular, has been shown to be associated with both master TFs and epigenetic chromatin marks. However, very little is known about how tissue-specific enhancers and, in particular, SEs emerge to support lineage commitment during development (38).

### Phase separated condensates formed at SEs

**Figure 3.**
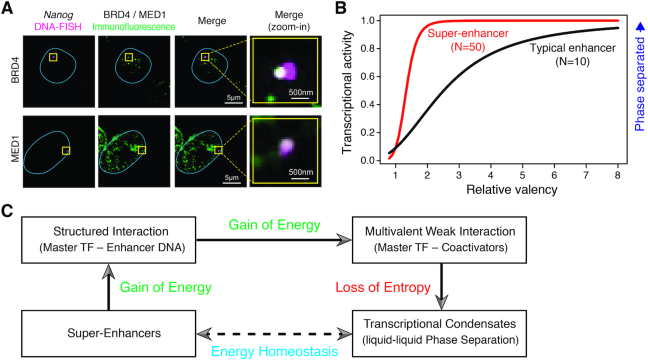
Phase-separated transcriptional condensates at super-enhancers. (**A**) Immunofluorescence and DNA-FISH showing the co-localization between BRD4 or MED1 condensates (magenta) and the *Nanog* gene locus (green) in fixed mouse ESCs. The blue lines indicate the nuclear boundaries, and the overlapping signal in the merged channels is shown in white (figure was adapted from (39) with permission from AAAS). (**B**) Theoretical curves showing the dependence of transcriptional activity on the increase of valency for SEs (parameterized as *N* = 50 molecules) and typical enhancers (*N* = 10) based on a phase-separation model proposed by Sharp *et al.* (50). While SEs reach a high transcriptional activity at relatively low valency, typical enhancers need higher valency to achieve the same transcriptional activity (figure was adapted from (50) with permission from Elsevier). (**C**) A schematic chart showing the energy homeostasis in formation of LLPS at SEs. Structured Interaction and multivalent interaction occur, providing energy to compensate the loss of entropy when LLPS forms and the system gets ordered.

SEs are extraordinarily densely bound by master TFs and highly occupied by the Mediator complex and other transcriptional coactivators, which raises the very interesting possibility that the genome can generate unusual physicochemical properties at their site of action. For example the Young lab has recently shown that the transcriptional coactivators BRD4 and MED1 are components of the liquid-liquid phase separated transcriptional condensates (39,40). They provided additional evidence that BRD4 and MED1 condensates co-localized with SEs (39) (Figure 3A). In a parallel study, Cho *et al.* showed further evidence that mediator and Pol II were co-existing in stable subcellular compartments, forming condensates and associated with SE elements (41). In both studies it was demonstrated that the formation of phase-separated liquid condensates were impaired in cells treated with 1,6-hexanediol, a compound known to disrupt these complexes (39,41). In addition, ChIP-seq with antibodies against BRD4 and MED1 revealed that the treatment of 1,6-hexanediol resulted in reduced BRD4 and MED1 binding at enhancers, and the effect was more profound at SEs (39). Mechanistically, SE-bound proteins such as BRD4 and MED1 typically contain large intrinsically disordered regions (IDRs), which multivalently but weakly interact with a large number of TFs and cofactors that also contain IDRs (40,42,43). The multi-valent interaction between IDRs facilitates condensation and liquid–liquid phase separation (LLPS) (39,44,45), where the highly concentrated transcriptional machinery guarantees the robust expression of cell identity genes. In support of this observation, Gibson *et al.* experimentally observed that adding BRD4 promoted formation of a new liquid phase of acetylated chromatin (46).

This is in concordance with related work in the field suggesting that the phase separation process plays a prominent role in 3D genome organization and is involved in organizing cell identity (47). The involvement of SEs in formation of LLPS also supports the assertion that SEs contribute more to transcriptional regulation than the additive effect of its multiple component TEs. More broadly it has been suggested that membraneless cellular organelles formed via phase separation play critical roles in a variety of cellular processes (reviewed in (48)). The localized protein concentration in these compartments is thought to be vital for the formation of phase-separated liquid droplets (49). The hub-like characteristics of SEs make the ideal biomolecular substrates to form phase-separated condensates comprising highly cooperative TFs, chromatin remodelers, transcription co-activators and RNA Pol II that elevate the local density ∼10-fold the molecular density of the components at typical enhancers (6–8). As a result, Sharp and colleagues hypothesized that formation of a phase separated assembly more likely occurred at SEs than at TEs (50). Based on this hypothesis, they proposed a kinetic model of transcriptional control, and explored the dynamic property of transcriptional activity by varying the number (*N*) of interacting molecules (i.e. the enhancer element and its associated factors) in a fixed-volume system, and the valency of each molecule. In this example, the transcriptional activity was quantitatively approximated by the relative size of the largest molecule cluster connected via cross-linking interactions (i.e. maximum size of clusters/*N*). When the transcriptional activity was reaching the value of 1, all molecules in the system formed a single interacting cluster, very likely resulting in the phase-separated assembly (50). By modeling SEs as a system containing 50 molecules while typical enhancers as a system consisting of 10 molecules, they revealed a divergent relationship between transcriptional activity and the change of valency between the two systems (Figure 3B). Where SEs reached a high transcriptional activity at low valency, typical enhancers needed higher valency to achieve the same transcriptional activity. This result suggests that SEs may undergo phase separation at a lower level of valency than typical enhancers. Moreover, a steeper increase of transcriptional activity of SEs was observed (Figure 3B), indicating that SEs likely behaved as binary switch in regulating key gene expression and could rapidly facilitate the establishment and maintenance of cell identity. In addition, the model also successfully explained a number of important observations in enhancer-mediated transcriptional control, including the transitional bursting patterns of enhancers and the ability of SEs to simultaneously activate multiple genes (50).

In terms of energy homeostasis, Chakraborty *et al.* claimed that formation of LLPS drives the chromatin in order and therefore leads to loss of entropy. This biophysical change requires compensation from energetic gain to maintain stable condensates. Where these form at enhancers, there are two ways to achieve this: (i) strong structured covalent master TF–DNA interaction and (ii) weak multivalent protein–protein (mostly TF-coactivators) interactions via IDRs (51). Therefore, SEs, exceeding other regulatory elements in both master TF binding sites and co-activator concentration, are more prone to the formation of transcriptional condensates (Figure 3C).

### 3D genome organization concerning SEs and their targets

The 3D genome organization has been shown to play critical roles in gene regulation and cell functions, also exhibiting cell-type specificity (47,52,53). In particular, insulated neighborhoods within chromosomal loop structures formed by CTCF-mediated interaction of two chromatin domains, provide a powerful paradigm for precise gene expression control (54). In other words, the superior transcriptional activity of SEs has to be strictly restricted within insulated neighborhoods such that they can be precisely and specifically tethered to their target genes. Even compared to stretch enhancers, relatively higher occupancy of cohesin and CTCF, the two factors mediating long-range DNA interaction and looping, has been found in constituents of SEs (29), supporting the notion that the chromosomal loops connecting SEs and promoters are more strictly controlled and maintained. An important question related to this is how the cell identity genes and their associated SEs are organized throughout the entire genome at the nucleotide sequence level?

**Figure 4.**
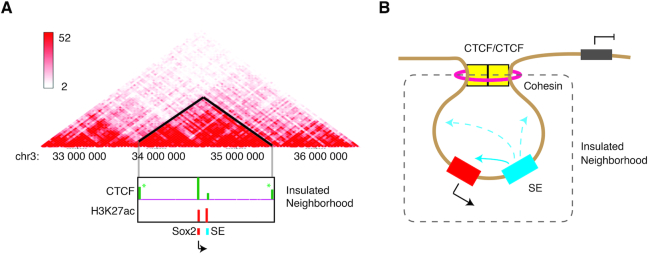
Chromatin 3D Organization and Insulated Neighborhoods. (**A**) Comparison of topologically associating domain (TAD) structure observed by Hi-C and insulated neighborhoods organized by two CTCF binding at borders. Upper panel shows the classical Hi-C interaction heatmap. Darker color indicates stronger normalized interaction frequency, represented by color key on the left side. Lower panel shows ChIP-seq of CTCF and H3K27ac within one TAD structure that encompasses *Sox2* and its SE. Note that CTCF binds to both sides of TAD boundaries (asterisk) which is also borders of insulated neighborhoods. The Hi-C data used for generating this plot were taken from Yan *et al.* (59). (**B**) A model cartoon showing the organization of insulated neighborhood. An SE (blue) and its target gene (red) are demarcated in a segment bordered by CTCF and associated cohesin complex. Due to the insulated environment, genes outside the neighborhood keep silent while inside genes are activated by the SEs.

Human genome contains many gene poor regions, called gene deserts that range in size from 5% to 40% of the entire chromosome (55). These segments often referred to as ‘junk DNA’ comprising 716 Mb, can be classified into two different categories based on their sequence conservation: stable gene deserts (>2% conserved) and variable gene deserts (<2% conserved). Intriguingly, gene ontology (GO) analysis shows that the tissue specific or developmentally regulated genes are moderately expressed and most of them are located in the stable gene deserts (55,56). In addition to the key cell identity gene bodies, their *cis*-regulatory elements are also embedded throughout the gene deserts. Comparative analysis has revealed that the density of transcriptional regulatory elements is three times higher in stable gene deserts than variable gene deserts and other intergenic regions (55). These *cis*-regulatory elements are generally linked to the neighboring genes, supported by a substantial depletion of synteny breakpoints in between. For example, the murine ESC pluripotent gene *Sox2* locus at chromosome 3qA3 is flanked by 1.5 Mb stable gene deserts and regulated by its SE 130 kb downstream of the gene body in the same gene desert (57,58,59) (Figure 4A). Another case is the human proto-oncogene *MYC* locus, which is located within a 1.2 Mb stable gene deserts at chromosome 8q24, regulated by a few distinct SEs in different tissues. Genome-wide association studies (GWAS) of multiple cancer and metabolic disease cohorts have identified single nucleotide polymorphisms (SNPs) in the gene deserts tightly linked to high risks of breast cancer, colorectal cancer, prostate cancer, ovarian cancer and so on (summarized in (60)). Some of these SNPs are located inside TF binding clusters (21), which are recognized as constituents of *MYC* gene SEs (8,32,61). A few more gene loci with similar genome arrangement have been reported, including loci of *KLF4*, *OTX2* and *DACH1* (62–64).

These observations suggest that the key genes tend to be independently organized from other chromosomal territories, which is likely to be evolutionarily favored for two non-mutually exclusive reasons, if not more: (i) enclosed genes are essential for cell identity and function, and thus require precise expression without interference by other signals other than its own SEs; (ii) SEs are so powerful that they need to be prevented from driving unrelated neighboring genes unexpectedly. Interestingly, in ESCs, Dowen *et al.* found that these cell identity genes not only occurred in gene deserts but also were restricted within insulated neighborhoods enclosed by CTCF and its associated cohesin, termed as SE domains (SDs) (65). The same phenomenon was described in a study on T-lymphoblastic leukemia (TLL) cells where an SE was restricted within the same CTCF-organized neighborhood of its target locus *IL7R*, a key gene for normal T cell development and pathogenesis of TLL (66). Deleting a CTCF binding site at one border of the SD caused dysregulation of internal cell identity genes and activation of external genes nearby the SDs (64,65). Disruption of insulated chromatin neighborhoods set for repressing proto-oncogene expression could activate oncogene and lead to severe health problems, further supporting a role of SD for precise control of essential gene expression (67).

The transcriptionally insulated neighborhoods, at a median size of ca. 200 kb, are formed by flanked CTCF binding in convergent orientations (64,65) (Figure 4B). Similar genome organization is observed as ‘contact domains’ (median size: 180 kb) identified from deeply sequenced *in situ* Hi-C data (68). The ‘contact domains’ are associated with distinct patterns of histone modifications across the domain borders. Taken together, this strongly deduces that ‘contact domains’ (by *in situ* Hi-C) and ‘insulated neighborhoods’ (by ChIA-PET) may refer to the same 3D chromatin structure units or at least with partial overlapping (64). When comparing the Hi-C data and ChIA-PET pairs carried out in the same primed human ESC (H1) line, Ji *et al.* found that the insulated neighborhoods were the fundamental units (subdomains) that constituted the megabase-sized topologically associating domains (TADs), which was also mediated by cohesin-associated CTCF–CTCF loops. Thus, they propose a model that genome is partitioned into many large and physically close TADs, which are constituted by multiple transcriptionally insulated neighborhoods. These subdomains delimit the effective range of enhancers, SEs and repressors (64).

However, forming the 3D genome configuration is a complex and dynamic process, which has yet to be completely understood (69). With combined efforts from multiple disciplines, a few models of transcriptional regulation and genome organization have been proposed. For example, a recent model based on physical principles suggests that forming clusters of active RNA polymerase and associated TFs is the elementary feature of 3D chromatin structures. These clusters are surrounded by DNA loops, and thus the large domains, TADs and chromatin A/B compartments are simply a single or multiple cluster with loops. This model also explains the extraordinary activity of SEs in transcriptional regulation, because Mediator and master TFs bound to SEs would increase the time of an associated promoter staying closely to the active polymerase clusters (70).

### A multi-stage transition model of the SE formation

Experimental evidence supports that binding of master TFs not only attracts more TFs, but also facilitates the recruitment of chromatin remodelers and transcriptional co-activators that possess enzymatic activity for histone modifications (71–76). Early biochemical studies uncovered that a single TF, C/EBP β, could compete against histones for DNA binding and mono-nucleosome formation (77), which was also confirmed at the chromatin level. A single master TF, HNF3 or GATA4, could compete against assembled nucleosome arrays (chromatin fibers) with assistance from its C-terminal DNA binding domain, and consequently makes the DNA accessible to other proteins including chromatin remodelers and modification enzymes (78). In a recent study, it has been found that immediately after DNA replication in each cell cycle, nucleosomes are repositioned at promoters and enhancers, followed by re-establishment of DNA accessibility led by TFs and chromatin remodelers in a fast mode (accomplished in hours) (79). As a result, phase-separated ‘transcription factories’ are formed around the protein-crowded chromatin foci, which can also be stably inherited along cellular division (80–83). Such ‘factory’ organization greatly increases the efficiency for multiple genes being coordinately regulated, and is supported by the recent observation that promoter-promoter interaction takes up 42% of total long-range interactions (84).

In some cases, one TF may be insufficient for nucleosome exclusion and chromatin opening, requiring instead cooperativity of multiple master TFs (85–87). For example, temporally persistent hierarchical binding of SOX2/OCT4/KLF4 prior to c-MYC was found in mammalian ESCs (87). Despite the importance of a cluster of TF binding for enhancer activity, the TF–DNA binding itself remains short-lived and highly dynamic. Chen *et al.* took advantage of super-resolution microscopical single-molecule tracking technology (SMT) and observed that TFs employed a 3D diffusion-dominated searching mode assisted by 1D sliding to locate their targeting sites, which took over 6 min (88). By contrast, the residential time for SOX2 binding to its stable recognition site lasted for only ∼12–14 s. These results suggest that TF binding alone could not fully explain the establishment of enhancers, whereas more stable and inheritable events are required, although TF binding *per se* is an initial step.

**Figure 5.**
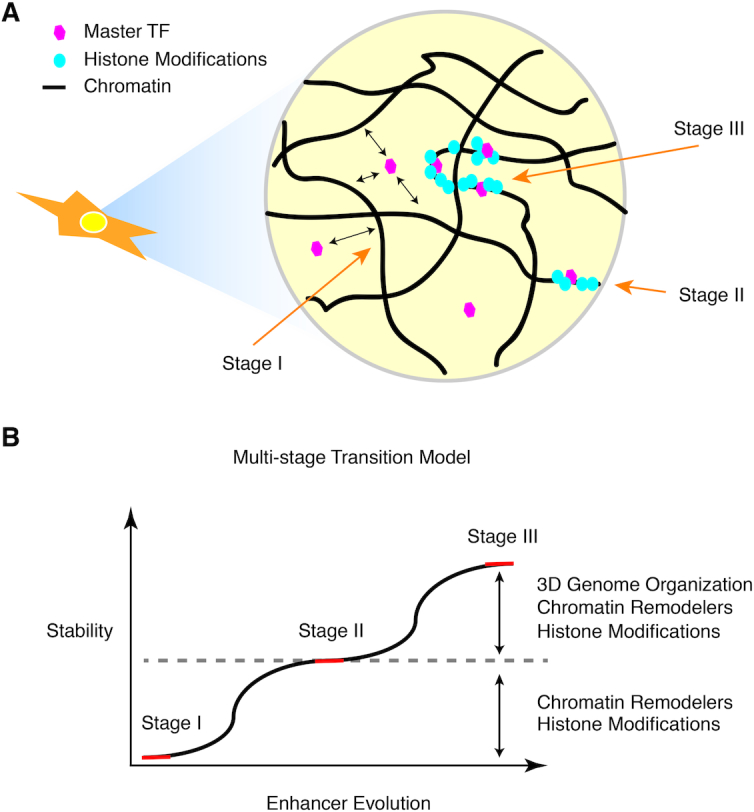
Multi-stage transition model of super-enhancer formation. (**A**) A schematic cartoon showing different stages of SE formation. Stage I: dynamic binding of the master TF; Stage II: accumulation of histone modifications and other TFs and stabilization of typical enhancers; Stage III: convergent evolution of synergistic typical enhancers to form SEs. (**B**) A diagram showing the process of enhancer evolution, represented in (A). Red line indicates the steady states while the black curve indicates the transition stages. Note that earlier stages are less stable than later stages where the enhancer is becoming larger in size and stronger in various protein binding signals and histone biochemical modifications.

Collectively, we introduce a ‘multi-stage transition’ model to describe the consecutive drift of evolution of the regulatory landscape from TF–DNA binding to SE formation (Figure 5). The initiation of the regulatory element takes place when a master TF searches for its target site, and competes against local nucleosomes for stable binding (Stage I). To achieve a more stable stage, such an event must be followed by recruitment of chromatin remodelers and histone modification enzymes to re-organize and maintain the more accessible and inheritable chromatin environment (Stage II). Along the evolution of cellular function, a few key genes demand synergistic regulation from multiple convergent regulatory elements and thus drive the formation of SEs (Stage III), the final destination for active transformation of *cis*-regulatory components (Figure 5).

A variety of evolutionary pressures separate the formation of SEs from TEs in different stages of the model. During Stages I and II, stable binding of a master TF is required initially to compete with nucleosomes, this is followed by the subsequent binding of other transcriptional activators and chromatin remodelers. As shown before in other examples, master TF binding at TEs is far less prevalent than at SEs. The lack of abundant master TF binding leads to a reduced enrichment of chromatin remodelers and co-activators (Stage II). The binding of BRD4 can facilitate the formation of LLPS between acetylated and un-acetylated chromatin (46). Upon establishment of LLPS, the nascent SEs become more stable than other foci. In the last stage, selection pressure eventually ‘selects’ the most functional SEs: (i) if a gene driven by a new SE is toxic to the cells, the corresponding SE hence becomes deleterious and will experience negatively selection and will tend to be removed from the cell population; (ii) when a gene driven by an SE is non-essential for cell function, the neutral genetic drift will dilute its presence in a cell population. Therefore, only the key genes that equip cells with proper functions will be positively preserved and retain their corresponding SEs during evolution. Analogously, a selective process could help oncogenes quickly develop SEs that predisposes them to uncontrolled growth advantage, and suggests a mechanism that could drive tumor cells to acquire SEs in tumor pathogenesis (8).

## SUPER-ENHANCERS AND DISEASES

SEs and their defects have been linked to multiple genetic diseases (8), including cancer (7,89), metabolic (8,90,91) and immune diseases (92) (reviewed in (93)). In this section, we will discuss current knowledge of SE-associated human health conditions, and the potential applications for disease diagnosis, prognosis, and treatment.

The vast majority of risk SNPs that confer genetic diseases rarely alter protein coding but mostly reside in non-coding regulatory loci (94,95). Significantly larger disease-associated mutation repertoire has been found in SEs compared to other open chromatin regions, such as promoters and typical enhancers (8). In some cases, small mutations and indels have been found to unexpectedly generate new SEs or rewire SEs to oncogenes that drive tumor pathogenesis (8,67,89,96).

### Cancers

SEs possess the capability of not only specifying cell identity but also maintaining cancer cell identity and discriminating carcinoma subtypes (97). The application of SE analysis to medulloblastoma is one of the first cases showing efficient way of using epigenetic data to trace the cellular origin of poorly characterized cancer malignancies (98,99). By querying their respective SEs together with their associated master TFs, Lin *et al.* managed to (i) locate the novel targets of the aberrant transcriptional system in different cancer subtypes and (ii) more importantly, identify cell-of-origin for cancer subgroups.

**Figure 6.**
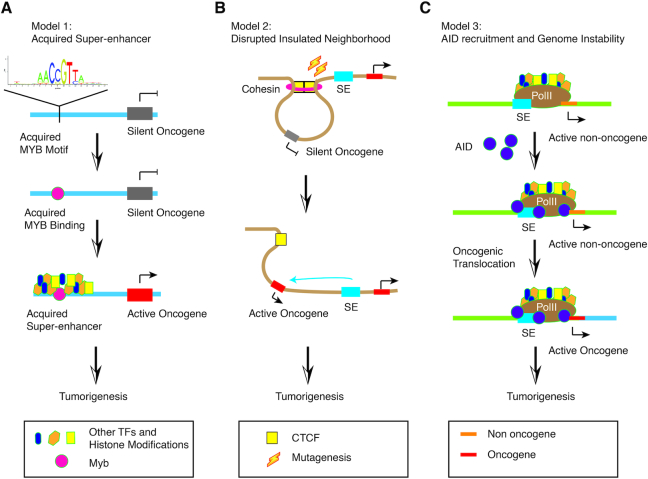
Three models of cancer-acquired super-enhancers. (**A**) Model 1: Due to mutation occurrence, a MYB binding motif is generated next to a silent oncogene, and the motif recruits MYB protein binding. Following the multi-stage transition model, the master TF binding accumulates more stable signals of other TFs, chromatin remodelers and histone modifications, resulting in formation of a stable and strong SE. Hence, the adjacent oncogene is activated (black arrow) by the newly acquired SE and causes oncogenesis. (**B**) Model 2: Unlike Model 1, the mutation does not create a novel binding site for master TFs but erases a binding site of CTCF, which is also an anchor site for an insulated neighborhood (brown circle). The formerly silent oncogene is activated by a juxtaposed SE and consequently results in tumorigenesis. Note that CTCF/Cohesin binding site mutation is significantly enriched in cancer genome. (**C**) Model 3: The activation-induced cytidine deaminase (AID) is accumulated to the largely permissive chromatin surrounding an SE by convergent transcripts from enhancer RNA and mRNA transcribed from the SE and gene body, respectively. Binding of AID triggers instability of genome and concomitantly translocation, which brings an oncogene next to the SE and promotes lymphomagenesis.

Multiple cancer types show either a prominent mutation rate or distorted regulatory landscape at the SEs of driver oncogenes in disease-relevant cell types. One particularly interesting example was discovered in T-cell acute lymphoblastic leukemia (T-ALL). For a long time, a helix-loop-helix TF TAL1 has been associated with bi-allelic activation in some T-ALL patients, which could be caused by loss of function of an upstream repressive regulatory element (100). In addition to these individuals with bi-allelic ectopic expression, it was found that there were a substantial number of patients and cell lines carrying mono-allelic overexpression of TAL1 (100). When the same team revisited the T-ALL subgroup with mono-allelic activation of TAL1, they discovered that the aberrant allele acquired a 20-kb long SE accommodating binding sites for a few major leukemogenic TFs, including RUNX1, GATA-3 and TAL1 itself (96). Strikingly, this oncogenic alteration was simply caused simply by a 12 bp insertion which generated a new MYB binding site that did not exist in cells without gaining the SE (Figure 6A).

Mutation or insertions that inappropriately activate enhancers to reboot a repressed disease driver gene, is not a novel finding in the pathogenesis of malignancy. In fact it is particularly common at the *MYC* locus (101,102). Whole-genome sequencing analysis of the widely-studied human cervical cancer cell line HeLa uncovered an active genomic fragment from Human Papilloma Virus type 18 (HPV-18) insertion into an originally silent constituent of a *MYC* super-enhancer, and thereafter drove upregulation of the proto-oncogene *MYC* promoting oncogenesis (103). However, in the TAL1 example, the short insertion for the *de novo* MYB binding site that creates a broad SE is rather unexpected. MYB binding itself is dynamic and thus may not be able to boost the activation of TAL1 expression. However, MYB, as a pioneer factor, draws chromatin remodelers, coactivators and other TFs in to establish a relatively stable regulatory niche. Accumulation of more and more executive activation factors to the mutated locus promotes formation of an SE that is powerful enough to drive oncogenic TAL1 expression and subsequent carcinogenesis. This model provides a novel mechanism for tumorigenesis through gaining binding sites of master TFs, which results in greater-than-expected gene expression and upregulation of oncogenes.

Cancers associated mutations are also frequently detected at CTCF and cohesin bound sequences, which are also enriched in SEs (104). With the whole genome sequencing of 231 colorectal cancer (CRC) patient tumors and paired normal intestinal tissues, Katainen *et al.* reported overall elevated mutation frequency in CTCF binding sites (CBSs) by an order of magnitude compared to average genome. Through further scrutiny of public cancer genome databases, they found that the CBSs were the somatic mutational hotspots in the non-coding cancer genome of multiple tumor types. These mutated CBSs were highly overlapping with CTCF-CTCF anchor sites that formed insulated neighborhoods in human ESCs. This is also consistent with another study by Hnisz *et al.* in which microdeletions caused loss of the boundary CTCF binding and consequently defeated the insulated neighborhood for repressed oncogenes expression in T-ALL (67) (Figure 6B).

During the process of somatic hypermutation and class-switch recombination, B cells have to undergo a series of DNA double strand breakage and highly frequent mutations triggered by activation-induced deaminase (AID) (105). Such a process, can aberrantly cause genome instability and malignancies. AID, which does not have any DNA sequencing binding motifs, has been shown with a high binding rate at extensively permissive SEs and transcriptionally active gene bodies (106,107). Comprehensive analyses dissecting the molecular mechanism involved show that Pol II stalled by convergent transcription collision recruits off-targeted AID to the largely permissive environment of SEs, which subsequently initiates mutations and translocations. Translocation can bring proto-oncogenes under the control of SEs resulting in over-expression and B cell lymphomagenesis (108) (Figure 6C).

### Developmental defects

Besides cancer, the proto-oncogene *MYC* is also linked to various developmental defects including common congenital malfunction, cleft lip with or without cleft palate (CL/P) (109–111). The *MYC* locus synteny is conserved between human and mouse. More interestingly, most of the mutation hotspots are enriched in cell type specific H3K27ac peaks, representing tissue-specific *MYC* enhancers and SEs (102,112–114). Using genome editing tools to manipulate the mouse *Myc* locus, Uslu *et al.* confirmed that a 300-kb medionasal super-enhancer (MNE) was responsible for 30% of *Myc* expression in face and limb during development, but not in liver or heart (102).

In comparison, manipulation of a typical enhancer may only have a marginal effect, considering its low regulatory activity (58,59,113,115). Thus, deletion of larger regulatory elements like SEs could help in the detection of such effects and better understanding relevant mechanisms. Systematically genome-wide validation of SE function for development and diseases is intriguing. The fast development of genome editing tool CRISPR/Cas9 (116,117) makes *in vivo* validation studies more feasible and should be more broadly applied in the near future (57,58,115).

A recent work published by the Mundlos lab showed *in vivo* evidence from animal model that CTCF organized topology of 3D chromatin structure was responsible for SE functions (118). Firstly, they identified three heterozygous deletions on neighboring chromosome regions in patients with three different limb genetic abnormalities – brachydactyly, F-syndrome and polydactyly. Subsequently, they used the CRISPR/Cas9 editing tool to reconstruct the chromosome deletions and successfully reproduced the disease phenotypes in mouse models. A 150-kb SE fragment was found to be the target as disruption of chromatin structure by deletion, inversion or duplication rewired this regulatory element to genes in neighboring TAD structure, which was originally insulated in wild-type cells. This mechanism is very similar to what was observed for oncogenes TAL1 and LMO2 in T-ALL as TAD structure itself is an insulated neighborhood (100).

### Potential therapeutic targets

Since SEs are heavily modified biochemically by various enzymes such as acetyltransferases or methyltransferases, they exhibit the potential ‘drug targetable’ characteristics. Two unique characteristics of SEs that we discussed above further make them particularly relevant targets for cancer therapy: (i) SEs are more sensitive to external signals than any other genomic regions; (ii) SEs are controlling cell identity genes in both healthy and disease cells. Both features could potentially guard the specificity of drugs, one of the most challenging issues in cancer therapy.

The epigenetic modification enzymes are found mutated at high frequency in cancer patients (∼20–40%), such as DNMT3A (DNA cytosine *de novo* methyltransferase 3A) in AML and MDS, and CBP/EP300 (lysine acetyltransferases) in bladder cancer and lymphomas (reviewed in (119)). The DNMT inhibitors, 5-azacytidine and decitabine, have been clinically used to effectively treat high risk myelodysplastic syndrome and AML (120). Among others, inhibitors of histone lysine deacetylases (HDACs), acetyltransferases (CBP) and chromatin remodelers are all available in the pharmaceutical market for cancer treatment (119). In addition to histone modification enzymes, targeting transcription coactivators is another feasible therapeutic strategy. Drugs such as JQ1 and THZ1, inhibiting BRD4 (7) and CDK7 (121), respectively, have been shown to specifically target tumor-specific SEs, providing an efficient way for targeting only cancer cells.

#### BRD4 and JQ1

The first compound reported to target SEs is ‘JQ1’, designed to specifically inhibit Bromodomain and Extraterminal (BET) superfamily member BRD4 that highly occupies SEs in cancer cells (7,122,123). BRD4 is known as a Mediator-interacting partner since the 1990s (124) and it is also a drug target in leukemia (125). Treating multiple myeloma tumor cells with JQ1 causes disproportional loss of BRD4 binding to the genome, more pronounced at SEs than TEs and other regions (7). Therefore, the SE controlled genes that include mostly oncogenes in cancer such as *MYC*, get more affected than other genes in tumor cells. Following independent studies, using JQ1 to target BRD4 and SEs, have demonstrated similar effects in a broad spectrum of cancer types, such as colorectal cancer (126), ovarian cancer (127), Merkel cell carcinoma (128), B cell lymphoma (129), and alveolar rhabdomyosarcoma (123). Of note, JQ1 is currently still being evaluated in phase I and II clinical trials, and resistance to JQ1 has been reported in a few cancer cases (130–132).

#### CDK7 and THZ1

Mediator super-enrichment in SEs also contributes to accumulation of Pol II holoenzyme and other subunits of transcription machinery. Cyclin Dependent Kinase 7 (CDK7) is a kinase subunit of a general transcription factor TFIIH, required for transcription machinery assembly. It can phosphorylate C-terminal domain (CTD) of Pol II and facilitate transcription initiation. Targeting CDK7 by its small molecule covalent inhibitor THZ1, can effectively inhibit master TF RUNX1 expression in T-ALL by disrupting its SE-associated transcription regulation circuitry (121). THZ1 shows a strong inhibitory effect on tumor growth in a human xenograft mouse model in a dose dependent manner. Similarly, THZ1 downregulates amplified MYCN expression in a high-risk neuroblastoma mouse model, notably, without any systemic toxicity (133). Strikingly, when the same strategy is applied to small-cell lung cancer (SCLC), on which there has been no significant therapeutic progress since chemotherapy was introduced in the 1970s, SE-associated genes, including MYC family genes, are highly vulnerable to the THZ1 treatment (134).

These two examples show that SE-associated protein factors are ideal options for drug targets. Besides the recent advances in genome editing technologies, CRISPR/Cas9, in particular, offer a possibility of novel gene therapies that directly correct pathogenic SEs (93). Of note, not only SEs and their directly interacting factors, but the pathways and master genes identified associated with disease-specific SEs also provide potential therapeutic targets, like transcription factors LMX1A, EOMES and LHX2 for group 4 medulloblastoma (98,99).

## CONCLUSIONS

In this Survey and Summary, we have highlighted the unique characteristics of SEs and their biological role in transcriptional regulation in health and disease. In particular, we addressed the ‘elephant in the room’: are SEs clusters of additive enhancers or a novel type of synergistic regulatory element? To address this question, we provide multiple lines of additional evidence suggesting that they are highly likely to be a unique biological entity in transcriptional regulation. Firstly, this is supported by a number of distinct characteristics of SEs compared to other enhancers, including their sequence composition, genomic size, regulatory activity, proteins bound, and genes under their regulation (also reviewed previously (135)). One of the most striking features of SE loci to emerge recently is that they are likely to be isolated from other chromatin domains by forming an insulated nuclear compartment through liquid-liquid phase-separated, membraneless condensates. In these focused compartments, they are more likely to have the necessary autonomy to precisely drive the regulation of genes controlling cell identity (40,136). Secondly, knockout experiments targeting SE components, furthermore demonstrate that functional hierarchy and synergistic interactions exist among different constituents within an SE (32,137). For instance, in a study targeting the constituents of the α-globin gene-associated SE (11), a linear-logistic model, which allowed for interactions between constituent enhancers, explained the knockout results better than a simple linear model (12), suggesting that the regulatory relationship between individual constituents are ‘synergistic’. Finally, SE constituents display the tissue specificity as an entire group. For example, SEs with multiple constituents in *MYC*-locus are located in a non-overlapping manner among different cell types (8,115): CRC tumors generally harbor an SE of MYC ∼300 kb upstream of its promoter, while in acute leukemia an SE 1.7 Mb downstream of the TSS plays a primary role of activating c-MYC expression (138,139). These observations further suggest the synergistic role of SE constituents in regulating gene expression. We therefore conclude that SEs form a distinct regulatory entity beyond additive clustering of independent enhancers, although more *in vivo* evidence is still required to further support this concept. Given the involvement of SEs in human pathology, future studies that generate greater understanding of their function as holistic entities, will be important for the development of new biomarkers and treatments that target these powerful genomic regulatory structures.

## DATA AVAILABILITY

A core collection of SEs used for the meta-analyses in this Survey and Summary and the computational scripts are available in the GitHub repository (https://sunlightwang.github.io/Super-Enhancers/).

## Supplementary Material

gkz1038_Supplemental_Files

## References

[1] Banerji J., Rusconi S., Schaffner W. Expression of a beta-globin gene is enhanced by remote SV40 DNA sequences. Cell. 1981; 27:299–308. 6277502 10.1016/0092-8674(81)90413-x

[2] Mercola M., Wang X.F., Olsen J., Calame K. Transcriptional enhancer elements in the mouse immunoglobulin heavy chain locus. Science. 1983; 221:663–665. 6306772 10.1126/science.6306772

[3] Banerji J., Olson L., Schaffner W. A lymphocyte-specific cellular enhancer is located downstream of the joining region in immunoglobulin heavy chain genes. Cell. 1983; 33:729–740. 6409418 10.1016/0092-8674(83)90015-6

[4] Gillies S.D., Morrison S.L., Oi V.T., Tonegawa S. A tissue-specific transcription enhancer element is located in the major intron of a rearranged immunoglobulin heavy chain gene. Cell. 1983; 33:717–728. 6409417 10.1016/0092-8674(83)90014-4

[5] Chen Y., Yao B., Zhu Z., Yi Y., Lin X., Zhang Z., Shen G. A constitutive super-enhancer: homologous region 3 of Bombyx mori nucleopolyhedrovirus. Biochem. Biophys. Res. Commun. 2004; 318:1039–1044. 15147978 10.1016/j.bbrc.2004.04.136

[6] Whyte W.A., Orlando D.A., Hnisz D., Abraham B.J., Lin C.Y., Kagey M.H., Rahl P.B., Lee T.I., Young R.A. Master transcription factors and mediator establish super-enhancers at key cell identity genes. Cell. 2013; 153:307–319. 23582322 10.1016/j.cell.2013.03.035 PMC3653129

[7] Loven J., Hoke H.A., Lin C.Y., Lau A., Orlando D.A., Vakoc C.R., Bradner J.E., Lee T.I., Young R.A. Selective inhibition of tumor oncogenes by disruption of super-enhancers. Cell. 2013; 153:320–334. 23582323 10.1016/j.cell.2013.03.036 PMC3760967

[8] Hnisz D., Abraham B.J., Lee T.I., Lau A., Saint-Andre V., Sigova A.A., Hoke H.A., Young R.A. Super-enhancers in the control of cell identity and disease. Cell. 2013; 155:934–947. 24119843 10.1016/j.cell.2013.09.053 PMC3841062

[9] Roadmap Epigenomics C., Kundaje A., Meuleman W., Ernst J., Bilenky M., Yen A., Heravi-Moussavi A., Kheradpour P., Zhang Z., Wang J. et al. Integrative analysis of 111 reference human epigenomes. Nature. 2015; 518:317–330. 25693563 10.1038/nature14248 PMC4530010

[10] Consortium E.P. An integrated encyclopedia of DNA elements in the human genome. Nature. 2012; 489:57–74. 22955616 10.1038/nature11247 PMC3439153

[11] Hay D., Hughes J.R., Babbs C., Davies J.O.J., Graham B.J., Hanssen L., Kassouf M.T., Marieke Oudelaar A.M., Sharpe J.A., Suciu M.C. et al. Genetic dissection of the alpha-globin super-enhancer in vivo. Nat. Genet. 2016; 48:895–903. 27376235 10.1038/ng.3605 PMC5058437

[12] Dukler N., Gulko B., Huang Y.F., Siepel A. Is a super-enhancer greater than the sum of its parts. Nat. Genet. 2016; 49:2–3. 28029159 10.1038/ng.3759 PMC5379849

[13] Moorthy S.D., Davidson S., Shchuka V.M., Singh G., Malek-Gilani N., Langroudi L., Martchenko A., So V., Macpherson N.N., Mitchell J.A. Enhancers and super-enhancers have an equivalent regulatory role in embryonic stem cells through regulation of single or multiple genes. Genome Res. 2017; 27:246–258. 27895109 10.1101/gr.210930.116 PMC5287230

[14] Khan A., Zhang X. dbSUPER: a database of super-enhancers in mouse and human genome. Nucleic Acids Res. 2016; 44:D164–D171. 26438538 10.1093/nar/gkv1002 PMC4702767

[15] Wei Y., Zhang S., Shang S., Zhang B., Li S., Wang X., Wang F., Su J., Wu Q., Liu H. et al. SEA: a super-enhancer archive. Nucleic Acids Res. 2016; 44:D172–D179. 26578594 10.1093/nar/gkv1243 PMC4702879

[16] Jiang Y., Qian F., Bai X., Liu Y., Wang Q., Ai B., Han X., Shi S., Zhang J., Li X. et al. SEdb: a comprehensive human super-enhancer database. Nucleic Acids Res. 2019; 47:D235–D243. 30371817 10.1093/nar/gky1025 PMC6323980

[17] Parker S.C., Stitzel M.L., Taylor D.L., Orozco J.M., Erdos M.R., Akiyama J.A., van Bueren K.L., Chines P.S., Narisu N., Program N.C.S. et al. Chromatin stretch enhancer states drive cell-specific gene regulation and harbor human disease risk variants. Proc. Natl. Acad. Sci. U.S.A. 2013; 110:17921–17926. 24127591 10.1073/pnas.1317023110 PMC3816444

[18] Gosselin D., Link V.M., Romanoski C.E., Fonseca G.J., Eichenfield D.Z., Spann N.J., Stender J.D., Chun H.B., Garner H., Geissmann F. et al. Environment drives selection and function of enhancers controlling tissue-specific macrophage identities. Cell. 2014; 159:1327–1340. 25480297 10.1016/j.cell.2014.11.023 PMC4364385

[19] Lukk M., Kapushesky M., Nikkila J., Parkinson H., Goncalves A., Huber W., Ukkonen E., Brazma A. A global map of human gene expression. Nat. Biotechnol. 2010; 28:322–324. 20379172 10.1038/nbt0410-322 PMC2974261

[20] Levine M. Transcriptional enhancers in animal development and evolution. Curr. Biol. 2010; 20:R754–R763. 20833320 10.1016/j.cub.2010.06.070 PMC4280268

[21] Yan J., Enge M., Whitington T., Dave K., Liu J., Sur I., Schmierer B., Jolma A., Kivioja T., Taipale M. et al. Transcription factor binding in human cells occurs in dense clusters formed around cohesin anchor sites. Cell. 2013; 154:801–813. 23953112 10.1016/j.cell.2013.07.034

[22] Boyer L.A., Lee T.I., Cole M.F., Johnstone S.E., Levine S.S., Zucker J.P., Guenther M.G., Kumar R.M., Murray H.L., Jenner R.G. et al. Core transcriptional regulatory circuitry in human embryonic stem cells. Cell. 2005; 122:947–956. 16153702 10.1016/j.cell.2005.08.020 PMC3006442

[23] Moorman C., Sun L.V., Wang J., de Wit E., Talhout W., Ward L.D., Greil F., Lu X.J., White K.P., Bussemaker H.J. et al. Hotspots of transcription factor colocalization in the genome of Drosophila melanogaster. Proc. Natl. Acad. Sci. U.S.A. 2006; 103:12027–12032. 16880385 10.1073/pnas.0605003103 PMC1567692

[24] Roy S., Ernst J., Kharchenko P.V., Kheradpour P., Negre N., Eaton M.L., Landolin J.M., Bristow C.A., Ma L., Lin M.F. et al. Identification of functional elements and regulatory circuits by Drosophila modENCODE. Science. 2010; 330:1787–1797. 21177974 10.1126/science.1198374 PMC3192495

[25] Stanojevic D., Small S., Levine M. Regulation of a segmentation stripe by overlapping activators and repressors in the Drosophila embryo. Science. 1991; 254:1385–1387. 1683715 10.1126/science.1683715

[26] Liu Z., Merkurjev D., Yang F., Li W., Oh S., Friedman M.J., Song X., Zhang F., Ma Q., Ohgi K.A. et al. Enhancer activation requires trans-recruitment of a mega transcription factor complex. Cell. 2014; 159:358–373. 25303530 10.1016/j.cell.2014.08.027 PMC4465761

[27] Siersbaek R., Rabiee A., Nielsen R., Sidoli S., Traynor S., Loft A., La Cour Poulsen L., Rogowska-Wrzesinska A., Jensen O.N., Mandrup S. Transcription factor cooperativity in early adipogenic hotspots and super-enhancers. Cell Rep. 2014; 7:1443–1455. 24857652 10.1016/j.celrep.2014.04.042

[28] Saint-Andre V., Federation A.J., Lin C.Y., Abraham B.J., Reddy J., Lee T.I., Bradner J.E., Young R.A. Models of human core transcriptional regulatory circuitries. Genome Res. 2016; 26:385–396. 26843070 10.1101/gr.197590.115 PMC4772020

[29] Khan A., Mathelier A., Zhang X. Super-enhancers are transcriptionally more active and cell type-specific than stretch enhancers. Epigenetics. 2018; 13:910–922. 30169995 10.1080/15592294.2018.1514231 PMC6284781

[30] Adam R.C., Yang H., Rockowitz S., Larsen S.B., Nikolova M., Oristian D.S., Polak L., Kadaja M., Asare A., Zheng D. et al. Pioneer factors govern super-enhancer dynamics in stem cell plasticity and lineage choice. Nature. 2015; 521:366–370. 25799994 10.1038/nature14289 PMC4482136

[31] Brown J.D., Lin C.Y., Duan Q., Griffin G., Federation A.J., Paranal R.M., Bair S., Newton G., Lichtman A.H., Kung A.L. et al. NF-kappaB directs dynamic super enhancer formation in inflammation and atherogenesis. Mol. Cell. 2014; 56:219–231. 25263595 10.1016/j.molcel.2014.08.024 PMC4224636

[32] Hnisz D., Schuijers J., Lin C.Y., Weintraub A.S., Abraham B.J., Lee T.I., Bradner J.E., Young R.A. Convergence of developmental and oncogenic signaling pathways at transcriptional super-enhancers. Mol. Cell. 2015; 58:362–370. 25801169 10.1016/j.molcel.2015.02.014 PMC4402134

[33] Kagey M.H., Newman J.J., Bilodeau S., Zhan Y., Orlando D.A., van Berkum N.L., Ebmeier C.C., Goossens J., Rahl P.B., Levine S.S. et al. Mediator and cohesin connect gene expression and chromatin architecture. Nature. 2010; 467:430–435. 20720539 10.1038/nature09380 PMC2953795

[34] Allen B.L., Taatjes D.J. The Mediator complex: a central integrator of transcription. Nat. Rev. Mol. Cell Biol. 2015; 16:155–166. 25693131 10.1038/nrm3951 PMC4963239

[35] Davis J.A., Takagi Y., Kornberg R.D., Asturias F.A. Structure of the yeast RNA polymerase II holoenzyme: Mediator conformation and polymerase interaction. Mol. Cell. 2002; 10:409–415. 12191485 10.1016/s1097-2765(02)00598-1

[36] Holstege F.C., Jennings E.G., Wyrick J.J., Lee T.I., Hengartner C.J., Green M.R., Golub T.R., Lander E.S., Young R.A. Dissecting the regulatory circuitry of a eukaryotic genome. Cell. 1998; 95:717–728. 9845373 10.1016/s0092-8674(00)81641-4

[37] Browning D.F., Busby S.J. The regulation of bacterial transcription initiation. Nat. Rev. Microbiol. 2004; 2:57–65. 15035009 10.1038/nrmicro787

[38] Peng X.L., So K.K., He L., Zhao Y., Zhou J., Li Y., Yao M., Xu B., Zhang S., Yao H. et al. MyoD- and FoxO3-mediated hotspot interaction orchestrates super-enhancer activity during myogenic differentiation. Nucleic Acids Res. 2017; 45:8785–8805. 28575289 10.1093/nar/gkx488 PMC5587775

[39] Sabari B.R., Dall’Agnese A., Boija A., Klein I.A., Coffey E.L., Shrinivas K., Abraham B.J., Hannett N.M., Zamudio A.V., Manteiga J.C. et al. Coactivator condensation at super-enhancers links phase separation and gene control. Science. 2018; 361:eaar3958. 29930091 10.1126/science.aar3958 PMC6092193

[40] Boija A., Klein I.A., Sabari B.R., Dall’Agnese A., Coffey E.L., Zamudio A.V., Li C.H., Shrinivas K., Manteiga J.C., Hannett N.M. et al. Transcription factors activate genes through the phase-separation capacity of their activation domains. Cell. 2018; 175:1842–1855. 30449618 10.1016/j.cell.2018.10.042 PMC6295254

[41] Cho W.K., Spille J.H., Hecht M., Lee C., Li C., Grube V., Cisse I.I. Mediator and RNA polymerase II clusters associate in transcription-dependent condensates. Science. 2018; 361:412–415. 29930094 10.1126/science.aar4199 PMC6543815

[42] Liu J., Perumal N.B., Oldfield C.J., Su E.W., Uversky V.N., Dunker A.K. Intrinsic disorder in transcription factors. Biochemistry. 2006; 45:6873–6888. 16734424 10.1021/bi0602718 PMC2538555

[43] Staby L., O'Shea C., Willemoes M., Theisen F., Kragelund B.B., Skriver K. Eukaryotic transcription factors: paradigms of protein intrinsic disorder. Biochem. J. 2017; 474:2509–2532. 28701416 10.1042/BCJ20160631

[44] Chong S., Dugast-Darzacq C., Liu Z., Dong P., Dailey G.M., Cattoglio C., Heckert A., Banala S., Lavis L., Darzacq X. et al. Imaging dynamic and selective low-complexity domain interactions that control gene transcription. Science. 2018; 361:eaar2555. 29930090 10.1126/science.aar2555 PMC6961784

[45] Cramer P. Organization and regulation of gene transcription. Nature. 2019; 573:45–54. 31462772 10.1038/s41586-019-1517-4

[46] Gibson B.A., Doolittle L.K., Schneider M.W.G., Jensen L.E., Gamarra N., Henry L., Gerlich D.W., Redding S., Rosen M.K. Organization of chromatin by intrinsic and regulated phase separation. Cell. 2019; 179:470–484. 31543265 10.1016/j.cell.2019.08.037 PMC6778041

[47] Stadhouders R., Filion G.J., Graf T. Transcription factors and 3D genome conformation in cell-fate decisions. Nature. 2019; 569:345–354. 31092938 10.1038/s41586-019-1182-7

[48] Boeynaems S., Alberti S., Fawzi N.L., Mittag T., Polymenidou M., Rousseau F., Schymkowitz J., Shorter J., Wolozin B., Van Den Bosch L. et al. Protein phase separation: a new phase in cell biology. Trends Cell Biol. 2018; 28:420–435. 29602697 10.1016/j.tcb.2018.02.004 PMC6034118

[49] Alberti S. Phase separation in biology. Curr. Biol. 2017; 27:R1097–R1102. 29065286 10.1016/j.cub.2017.08.069

[50] Hnisz D., Shrinivas K., Young R.A., Chakraborty A.K., Sharp P.A. A phase separation model for transcriptional control. Cell. 2017; 169:13–23. 28340338 10.1016/j.cell.2017.02.007 PMC5432200

[51] Shrinivas K., Sabari B.R., Coffey E.L., Klein I.A., Boija A., Zamudio A.V., Schuijers J., Hannett N.M., Sharp P.A., Young R.A. et al. Enhancer features that drive formation of transcriptional condensates. Mol. Cell. 2019; 75:549–561. 31398323 10.1016/j.molcel.2019.07.009 PMC6690378

[52] Tan L., Xing D., Daley N., Xie X.S. Three-dimensional genome structures of single sensory neurons in mouse visual and olfactory systems. Nat. Struct. Mol. Biol. 2019; 26:297–307. 30936528 10.1038/s41594-019-0205-2

[53] Tan L., Xing D., Chang C.H., Li H., Xie X.S. Three-dimensional genome structures of single diploid human cells. Science. 2018; 361:924–928. 30166492 10.1126/science.aat5641 PMC6360088

[54] Hnisz D., Day D.S., Young R.A. Insulated neighborhoods: structural and functional units of mammalian gene control. Cell. 2016; 167:1188–1200. 27863240 10.1016/j.cell.2016.10.024 PMC5125522

[55] Ovcharenko I., Loots G.G., Nobrega M.A., Hardison R.C., Miller W., Stubbs L. Evolution and functional classification of vertebrate gene deserts. Genome Res. 2005; 15:137–145. 15590943 10.1101/gr.3015505 PMC540279

[56] Taylor J. Clues to function in gene deserts. Trends Biotechnol. 2005; 23:269–271. 15922077 10.1016/j.tibtech.2005.04.003

[57] Li Y., Rivera C.M., Ishii H., Jin F., Selvaraj S., Lee A.Y., Dixon J.R., Ren B. CRISPR reveals a distal super-enhancer required for Sox2 expression in mouse embryonic stem cells. PLoS One. 2014; 9:e114485. 25486255 10.1371/journal.pone.0114485 PMC4259346

[58] Zhou H.Y., Katsman Y., Dhaliwal N.K., Davidson S., Macpherson N.N., Sakthidevi M., Collura F., Mitchell J.A. A Sox2 distal enhancer cluster regulates embryonic stem cell differentiation potential. Genes Dev. 2014; 28:2699–2711. 25512558 10.1101/gad.248526.114 PMC4265674

[59] Yan J., Chen S.A., Local A., Liu T., Qiu Y., Dorighi K.M., Preissl S., Rivera C.M., Wang C., Ye Z. et al. Histone H3 lysine 4 monomethylation modulates long-range chromatin interactions at enhancers. Cell Res. 2018; 28:204–220. 29313530 10.1038/cr.2018.1 PMC5799818

[60] Ghoussaini M., Song H., Koessler T., Al Olama A.A., Kote-Jarai Z., Driver K.E., Pooley K.A., Ramus S.J., Kjaer S.K., Hogdall E. et al. Multiple loci with different cancer specificities within the 8q24 gene desert. J. Natl. Cancer Inst. 2008; 100:962–966. 18577746 10.1093/jnci/djn190 PMC2902819

[61] Heyn H., Vidal E., Ferreira H.J., Vizoso M., Sayols S., Gomez A., Moran S., Boque-Sastre R., Guil S., Martinez-Cardus A. et al. Epigenomic analysis detects aberrant super-enhancer DNA methylation in human cancer. Genome Biol. 2016; 17:11. 26813288 10.1186/s13059-016-0879-2 PMC4728783

[62] Nobrega M.A., Ovcharenko I., Afzal V., Rubin E.M. Scanning human gene deserts for long-range enhancers. Science. 2003; 302:413. 14563999 10.1126/science.1088328

[63] Kimura-Yoshida C., Kitajima K., Oda-Ishii I., Tian E., Suzuki M., Yamamoto M., Suzuki T., Kobayashi M., Aizawa S., Matsuo I. Characterization of the pufferfish Otx2 cis-regulators reveals evolutionarily conserved genetic mechanisms for vertebrate head specification. Development. 2004; 131:57–71. 14645121 10.1242/dev.00877

[64] Ji X., Dadon D.B., Powell B.E., Fan Z.P., Borges-Rivera D., Shachar S., Weintraub A.S., Hnisz D., Pegoraro G., Lee T.I. et al. 3D chromosome regulatory landscape of human pluripotent cells. Cell Stem Cell. 2016; 18:262–275. 26686465 10.1016/j.stem.2015.11.007 PMC4848748

[65] Dowen J.M., Fan Z.P., Hnisz D., Ren G., Abraham B.J., Zhang L.N., Weintraub A.S., Schuijers J., Lee T.I., Zhao K. et al. Control of cell identity genes occurs in insulated neighborhoods in mammalian chromosomes. Cell. 2014; 159:374–387. 25303531 10.1016/j.cell.2014.09.030 PMC4197132

[66] Wang H., Zang C., Taing L., Arnett K.L., Wong Y.J., Pear W.S., Blacklow S.C., Liu X.S., Aster J.C. NOTCH1-RBPJ complexes drive target gene expression through dynamic interactions with superenhancers. Proc. Natl. Acad. Sci. U.S.A. 2014; 111:705–710. 24374627 10.1073/pnas.1315023111 PMC3896193

[67] Hnisz D., Weintraub A.S., Day D.S., Valton A.L., Bak R.O., Li C.H., Goldmann J., Lajoie B.R., Fan Z.P., Sigova A.A. et al. Activation of proto-oncogenes by disruption of chromosome neighborhoods. Science. 2016; 351:1454–1458. 26940867 10.1126/science.aad9024 PMC4884612

[68] Rao S.S., Huntley M.H., Durand N.C., Stamenova E.K., Bochkov I.D., Robinson J.T., Sanborn A.L., Machol I., Omer A.D., Lander E.S. et al. A 3D map of the human genome at kilobase resolution reveals principles of chromatin looping. Cell. 2014; 159:1665–1680. 25497547 10.1016/j.cell.2014.11.021 PMC5635824

[69] Zheng H., Xie W. The role of 3D genome organization in development and cell differentiation. Nat. Rev. Mol. Cell Biol. 2019; 20:535–550. 31197269 10.1038/s41580-019-0132-4

[70] Cook P.R., Marenduzzo D. Transcription-driven genome organization: a model for chromosome structure and the regulation of gene expression tested through simulations. Nucleic Acids Res. 2018; 46:9895–9906. 30239812 10.1093/nar/gky763 PMC6212781

[71] Puri P.L., Sartorelli V., Yang X.J., Hamamori Y., Ogryzko V.V., Howard B.H., Kedes L., Wang J.Y., Graessmann A., Nakatani Y. et al. Differential roles of p300 and PCAF acetyltransferases in muscle differentiation. Mol. Cell. 1997; 1:35–45. 9659901 10.1016/s1097-2765(00)80005-2

[72] Rampalli S., Li L., Mak E., Ge K., Brand M., Tapscott S.J., Dilworth F.J. p38 MAPK signaling regulates recruitment of Ash2L-containing methyltransferase complexes to specific genes during differentiation. Nat. Struct. Mol. Biol. 2007; 14:1150–1156. 18026121 10.1038/nsmb1316 PMC4152845

[73] Simone C., Forcales S.V., Hill D.A., Imbalzano A.N., Latella L., Puri P.L. p38 pathway targets SWI-SNF chromatin-remodeling complex to muscle-specific loci. Nat. Genet. 2004; 36:738–743. 15208625 10.1038/ng1378

[74] Cao Y., Kumar R.M., Penn B.H., Berkes C.A., Kooperberg C., Boyer L.A., Young R.A., Tapscott S.J. Global and gene-specific analyses show distinct roles for Myod and Myog at a common set of promoters. EMBO J. 2006; 25:502–511. 16437161 10.1038/sj.emboj.7600958 PMC1383539

[75] Yuan W., Condorelli G., Caruso M., Felsani A., Giordano A. Human p300 protein is a coactivator for the transcription factor MyoD. J. Biol. Chem. 1996; 271:9009–9013. 8621548 10.1074/jbc.271.15.9009

[76] Blum R., Vethantham V., Bowman C., Rudnicki M., Dynlacht B.D. Genome-wide identification of enhancers in skeletal muscle: the role of MyoD1. Genes Dev. 2012; 26:2763–2779. 23249738 10.1101/gad.200113.112 PMC3533080

[77] Svaren J., Klebanow E., Sealy L., Chalkley R. Analysis of the competition between nucleosome formation and transcription factor binding. J. Biol. Chem. 1994; 269:9335–9344. 8132673

[78] Cirillo L.A., Lin F.R., Cuesta I., Friedman D., Jarnik M., Zaret K.S. Opening of compacted chromatin by early developmental transcription factors HNF3 (FoxA) and GATA-4. Mol. Cell. 2002; 9:279–289. 11864602 10.1016/s1097-2765(02)00459-8

[79] Ramachandran S., Henikoff S. Transcriptional regulators compete with nucleosomes post-replication. Cell. 2016; 165:580–592. 27062929 10.1016/j.cell.2016.02.062 PMC4855302

[80] Xu M., Wang W., Chen S., Zhu B. A model for mitotic inheritance of histone lysine methylation. EMBO Rep. 2012; 13:60–67. 10.1038/embor.2011.206 PMC3246248 22056817

[81] Audergon P.N., Catania S., Kagansky A., Tong P., Shukla M., Pidoux A.L., Allshire R.C. Epigenetics. Restricted epigenetic inheritance of H3K9 methylation. Science. 2015; 348:132–135. 25838386 10.1126/science.1260638 PMC4397586

[82] Ragunathan K., Jih G., Moazed D. Epigenetics. Epigenetic inheritance uncoupled from sequence-specific recruitment. Science. 2015; 348:1258699. 25831549 10.1126/science.1258699 PMC4385470

[83] Hattori N., Niwa T., Kimura K., Helin K., Ushijima T. Visualization of multivalent histone modification in a single cell reveals highly concerted epigenetic changes on differentiation of embryonic stem cells. Nucleic Acids Res. 2013; 41:7231–7239. 23761442 10.1093/nar/gkt528 PMC3753646

[84] Li G., Ruan X., Auerbach R.K., Sandhu K.S., Zheng M., Wang P., Poh H.M., Goh Y., Lim J., Zhang J. et al. Extensive promoter-centered chromatin interactions provide a topological basis for transcription regulation. Cell. 2012; 148:84–98. 22265404 10.1016/j.cell.2011.12.014 PMC3339270

[85] Moyle-Heyrman G., Tims H.S., Widom J. Structural constraints in collaborative competition of transcription factors against the nucleosome. J. Mol. Biol. 2011; 412:634–646. 21821044 10.1016/j.jmb.2011.07.032 PMC3534743

[86] Adams C.C., Workman J.L. Binding of disparate transcriptional activators to nucleosomal DNA is inherently cooperative. Mol. Cell Biol. 1995; 15:1405–1421. 7862134 10.1128/mcb.15.3.1405 PMC230365

[87] Soufi A., Garcia M.F., Jaroszewicz A., Osman N., Pellegrini M., Zaret K.S. Pioneer transcription factors target partial DNA motifs on nucleosomes to initiate reprogramming. Cell. 2015; 161:555–568. 25892221 10.1016/j.cell.2015.03.017 PMC4409934

[88] Chen J., Zhang Z., Li L., Chen B.C., Revyakin A., Hajj B., Legant W., Dahan M., Lionnet T., Betzig E. et al. Single-molecule dynamics of enhanceosome assembly in embryonic stem cells. Cell. 2014; 156:1274–1285. 24630727 10.1016/j.cell.2014.01.062 PMC4040518

[89] Vaharautio A., Taipale J. Cancer. Cancer by super-enhancer. Science. 2014; 346:1291–1292. 25504702 10.1126/science.aaa3247

[90] Ounzain S., Pedrazzini T. Super-enhancer lncs to cardiovascular development and disease. Biochim. Biophys. Acta. 2015; 1863:1953–1960. 26620798 10.1016/j.bbamcr.2015.11.026

[91] Pacheco M.P., John E., Kaoma T., Heinaniemi M., Nicot N., Vallar L., Bueb J.L., Sinkkonen L., Sauter T. Integrated metabolic modelling reveals cell-type specific epigenetic control points of the macrophage metabolic network. BMC Genomics. 2015; 16:809. 26480823 10.1186/s12864-015-1984-4 PMC4617894

[92] Herranz D., Ambesi-Impiombato A., Palomero T., Schnell S.A., Belver L., Wendorff A.A., Xu L., Castillo-Martin M., Llobet-Navas D., Cordon-Cardo C. et al. A NOTCH1-driven MYC enhancer promotes T cell development, transformation and acute lymphoblastic leukemia. Nat. Med. 2014; 20:1130–1137. 25194570 10.1038/nm.3665 PMC4192073

[93] Shin H.Y. Targeting Super-Enhancers for disease treatment and diagnosis. Mol. Cells. 2018; 41:506–514. 29754476 10.14348/molcells.2018.2297 PMC6030247

[94] Maurano M.T., Humbert R., Rynes E., Thurman R.E., Haugen E., Wang H., Reynolds A.P., Sandstrom R., Qu H., Brody J. et al. Systematic localization of common disease-associated variation in regulatory DNA. Science. 2012; 337:1190–1195. 22955828 10.1126/science.1222794 PMC3771521

[95] Tuupanen S., Yan J., Turunen M., Gylfe A.E., Kaasinen E., Li L., Eng C., Culver D.A., Kalady M.F., Pennison M.J. et al. Characterization of the colorectal cancer-associated enhancer MYC-335 at 8q24: the role of rs67491583. Cancer Genet. 2012; 205:25–33. 22429595 10.1016/j.cancergen.2012.01.005 PMC3770308

[96] Mansour M.R., Abraham B.J., Anders L., Berezovskaya A., Gutierrez A., Durbin A.D., Etchin J., Lawton L., Sallan S.E., Silverman L.B. et al. Oncogene regulation. An oncogenic super-enhancer formed through somatic mutation of a noncoding intergenic element. Science. 2014; 346:1373–1377. 25394790 10.1126/science.1259037 PMC4720521

[97] Sengupta S., George R.E. Super-enhancer-driven transcriptional dependencies in cancer. Trends Cancer. 2017; 3:269–281. 28718439 10.1016/j.trecan.2017.03.006 PMC5546010

[98] Lin C.Y., Erkek S., Tong Y., Yin L., Federation A.J., Zapatka M., Haldipur P., Kawauchi D., Risch T., Warnatz H.J. et al. Active medulloblastoma enhancers reveal subgroup-specific cellular origins. Nature. 2016; 530:57–62. 26814967 10.1038/nature16546 PMC5168934

[99] Northcott P.A., Korshunov A., Pfister S.M., Taylor M.D. The clinical implications of medulloblastoma subgroups. Nat. Rev. Neurol. 2012; 8:340–351. 22565209 10.1038/nrneurol.2012.78

[100] Ferrando A.A., Herblot S., Palomero T., Hansen M., Hoang T., Fox E.A., Look A.T. Biallelic transcriptional activation of oncogenic transcription factors in T-cell acute lymphoblastic leukemia. Blood. 2004; 103:1909–1911. 14604958 10.1182/blood-2003-07-2577

[101] Edelmann J., Holzmann K., Miller F., Winkler D., Buhler A., Zenz T., Bullinger L., Kuhn M.W., Gerhardinger A., Bloehdorn J. et al. High-resolution genomic profiling of chronic lymphocytic leukemia reveals new recurrent genomic alterations. Blood. 2012; 120:4783–4794. 23047824 10.1182/blood-2012-04-423517

[102] Uslu V.V., Petretich M., Ruf S., Langenfeld K., Fonseca N.A., Marioni J.C., Spitz F. Long-range enhancers regulating Myc expression are required for normal facial morphogenesis. Nat. Genet. 2014; 46:753–758. 24859337 10.1038/ng.2971

[103] Adey A., Burton J.N., Kitzman J.O., Hiatt J.B., Lewis A.P., Martin B.K., Qiu R., Lee C., Shendure J. The haplotype-resolved genome and epigenome of the aneuploid HeLa cancer cell line. Nature. 2013; 500:207–211. 23925245 10.1038/nature12064 PMC3740412

[104] Katainen R., Dave K., Pitkanen E., Palin K., Kivioja T., Valimaki N., Gylfe A.E., Ristolainen H., Hanninen U.A., Cajuso T. et al. CTCF/cohesin-binding sites are frequently mutated in cancer. Nat. Genet. 2015; 47:818–821. 26053496 10.1038/ng.3335

[105] Nussenzweig A., Nussenzweig M.C. Origin of chromosomal translocations in lymphoid cancer. Cell. 2010; 141:27–38. 20371343 10.1016/j.cell.2010.03.016 PMC2874895

[106] Meng F.L., Du Z., Federation A., Hu J., Wang Q., Kieffer-Kwon K.R., Meyers R.M., Amor C., Wasserman C.R., Neuberg D. et al. Convergent transcription at intragenic super-enhancers targets AID-initiated genomic instability. Cell. 2014; 159:1538–1548. 25483776 10.1016/j.cell.2014.11.014 PMC4322776

[107] Qian J., Wang Q., Dose M., Pruett N., Kieffer-Kwon K.R., Resch W., Liang G., Tang Z., Mathe E., Benner C. et al. B cell super-enhancers and regulatory clusters recruit AID tumorigenic activity. Cell. 2014; 159:1524–1537. 25483777 10.1016/j.cell.2014.11.013 PMC4272762

[108] Alt F.W., Zhang Y., Meng F.L., Guo C., Schwer B. Mechanisms of programmed DNA lesions and genomic instability in the immune system. Cell. 2013; 152:417–429. 23374339 10.1016/j.cell.2013.01.007 PMC4382911

[109] Nesbit C.E., Tersak J.M., Prochownik E.V. MYC oncogenes and human neoplastic disease. Oncogene. 1999; 18:3004–3016. 10378696 10.1038/sj.onc.1202746

[110] Jackson T., Allard M.F., Sreenan C.M., Doss L.K., Bishop S.P., Swain J.L. The c-myc proto-oncogene regulates cardiac development in transgenic mice. Mol. Cell Biol. 1990; 10:3709–3716. 1694017 10.1128/mcb.10.7.3709-3716.1990 PMC360819

[111] Ludwig K.U., Mangold E., Herms S., Nowak S., Reutter H., Paul A., Becker J., Herberz R., AlChawa T., Nasser E. et al. Genome-wide meta-analyses of nonsyndromic cleft lip with or without cleft palate identify six new risk loci. Nat. Genet. 2012; 44:968–971. 22863734 10.1038/ng.2360 PMC3598617

[112] Sotelo J., Esposito D., Duhagon M.A., Banfield K., Mehalko J., Liao H., Stephens R.M., Harris T.J., Munroe D.J., Wu X. Long-range enhancers on 8q24 regulate c-Myc. Proc. Natl. Acad. Sci. U.S.A. 2010; 107:3001–3005. 20133699 10.1073/pnas.0906067107 PMC2840341

[113] Sur I.K., Hallikas O., Vaharautio A., Yan J., Turunen M., Enge M., Taipale M., Karhu A., Aaltonen L.A., Taipale J. Mice lacking a Myc enhancer that includes human SNP rs6983267 are resistant to intestinal tumors. Science. 2012; 338:1360–1363. 23118011 10.1126/science.1228606

[114] Tuupanen S., Turunen M., Lehtonen R., Hallikas O., Vanharanta S., Kivioja T., Bjorklund M., Wei G., Yan J., Niittymaki I. et al. The common colorectal cancer predisposition SNP rs6983267 at chromosome 8q24 confers potential to enhanced Wnt signaling. Nat. Genet. 2009; 41:885–890. 19561604 10.1038/ng.406

[115] Dave K., Sur I., Yan J., Zhang J., Kaasinen E., Zhong F., Blaas L., Li X., Kharazi S., Gustafsson C. et al. Mice deficient of Myc super-enhancer region reveal differential control mechanism between normal and pathological growth. Elife. 2017; 6:e23382. 28583252 10.7554/eLife.23382 PMC5461110

[116] Cong L., Ran F.A., Cox D., Lin S., Barretto R., Habib N., Hsu P.D., Wu X., Jiang W., Marraffini L.A. et al. Multiplex genome engineering using CRISPR/Cas systems. Science. 2013; 339:819–823. 23287718 10.1126/science.1231143 PMC3795411

[117] Mali P., Yang L., Esvelt K.M., Aach J., Guell M., DiCarlo J.E., Norville J.E., Church G.M. RNA-guided human genome engineering via Cas9. Science. 2013; 339:823–826. 23287722 10.1126/science.1232033 PMC3712628

[118] Lupianez D.G., Kraft K., Heinrich V., Krawitz P., Brancati F., Klopocki E., Horn D., Kayserili H., Opitz J.M., Laxova R. et al. Disruptions of topological chromatin domains cause pathogenic rewiring of gene-enhancer interactions. Cell. 2015; 161:1012–1025. 25959774 10.1016/j.cell.2015.04.004 PMC4791538

[119] Geutjes E.J., Bajpe P.K., Bernards R. Targeting the epigenome for treatment of cancer. Oncogene. 2012; 31:3827–3844. 22139071 10.1038/onc.2011.552

[120] Kelly T.K., De Carvalho D.D., Jones P.A. Epigenetic modifications as therapeutic targets. Nat. Biotechnol. 2010; 28:1069–1078. 20944599 10.1038/nbt.1678 PMC3022972

[121] Kwiatkowski N., Zhang T., Rahl P.B., Abraham B.J., Reddy J., Ficarro S.B., Dastur A., Amzallag A., Ramaswamy S., Tesar B. et al. Targeting transcription regulation in cancer with a covalent CDK7 inhibitor. Nature. 2014; 511:616–620. 25043025 10.1038/nature13393 PMC4244910

[122] Anders L., Guenther M.G., Qi J., Fan Z.P., Marineau J.J., Rahl P.B., Loven J., Sigova A.A., Smith W.B., Lee T.I. et al. Genome-wide localization of small molecules. Nat. Biotechnol. 2014; 32:92–96. 24336317 10.1038/nbt.2776 PMC4189815

[123] Gryder B.E., Yohe M.E., Chou H.C., Zhang X., Marques J., Wachtel M., Schaefer B., Sen N., Song Y., Gualtieri A. et al. PAX3-FOXO1 establishes myogenic super enhancers and confers BET bromodomain vulnerability. Cancer Discov. 2017; 7:884–899. 28446439 10.1158/2159-8290.CD-16-1297 PMC7802885

[124] Jiang Y.W., Veschambre P., Erdjument-Bromage H., Tempst P., Conaway J.W., Conaway R.C., Kornberg R.D. Mammalian mediator of transcriptional regulation and its possible role as an end-point of signal transduction pathways. Proc. Natl. Acad. Sci. U.S.A. 1998; 95:8538–8543. 9671713 10.1073/pnas.95.15.8538 PMC21111

[125] Dawson M.A., Prinjha R.K., Dittmann A., Giotopoulos G., Bantscheff M., Chan W.I., Robson S.C., Chung C.W., Hopf C., Savitski M.M. et al. Inhibition of BET recruitment to chromatin as an effective treatment for MLL-fusion leukaemia. Nature. 2011; 478:529–533. 21964340 10.1038/nature10509 PMC3679520

[126] Togel L., Nightingale R., Chueh A.C., Jayachandran A., Tran H., Phesse T., Wu R., Sieber O.M., Arango D., Dhillon A.S. et al. Dual targeting of bromodomain and extra-terminal domain proteins, and WNT or MAPK signaling, inhibits c-MYC expression and proliferation of colorectal cancer cells. Mol. Cancer Ther. 2016; 15:1217–1226. 26983878 10.1158/1535-7163.MCT-15-0724

[127] Zhang Z., Ma P., Jing Y., Yan Y., Cai M.C., Zhang M., Zhang S., Peng H., Ji Z.L., Di W. et al. BET bromodomain inhibition as a therapeutic strategy in ovarian cancer by downregulating FoxM1. Theranostics. 2016; 6:219–230. 26877780 10.7150/thno.13178 PMC4729770

[128] Sengupta D., Kannan A., Kern M., Moreno M.A., Vural E., Stack B. Jr, Suen J.Y., Tackett A.J., Gao L. Disruption of BRD4 at H3K27Ac-enriched enhancer region correlates with decreased c-Myc expression in Merkel cell carcinoma. Epigenetics. 2015; 10:460–466. 25941994 10.1080/15592294.2015.1034416 PMC4622756

[129] Chapuy B., McKeown M.R., Lin C.Y., Monti S., Roemer M.G., Qi J., Rahl P.B., Sun H.H., Yeda K.T., Doench J.G. et al. Discovery and characterization of super-enhancer-associated dependencies in diffuse large B cell lymphoma. Cancer Cell. 2013; 24:777–790. 24332044 10.1016/j.ccr.2013.11.003 PMC4018722

[130] Pawar A., Gollavilli P.N., Wang S., Asangani I.A. Resistance to BET inhibitor leads to alternative therapeutic vulnerabilities in castration-resistant prostate cancer. Cell Rep. 2018; 22:2236–2245. 29490263 10.1016/j.celrep.2018.02.011

[131] Jin X., Yan Y., Wang D., Ding D., Ma T., Ye Z., Jimenez R., Wang L., Wu H., Huang H. DUB3 promotes bet inhibitor resistance and cancer progression by deubiquitinating BRD4. Mol. Cell. 2018; 71:592–605. 30057199 10.1016/j.molcel.2018.06.036 PMC6086352

[132] Yin Y., Sun M., Zhan X., Wu C., Geng P., Sun X., Wu Y., Zhang S., Qin J., Zhuang Z. et al. EGFR signaling confers resistance to BET inhibition in hepatocellular carcinoma through stabilizing oncogenic MYC. J. Exp. Clin. Cancer Res. 2019; 38:83. 30770740 10.1186/s13046-019-1082-6 PMC6377788

[133] Chipumuro E., Marco E., Christensen C.L., Kwiatkowski N., Zhang T., Hatheway C.M., Abraham B.J., Sharma B., Yeung C., Altabef A. et al. CDK7 inhibition suppresses super-enhancer-linked oncogenic transcription in MYCN-driven cancer. Cell. 2014; 159:1126–1139. 25416950 10.1016/j.cell.2014.10.024 PMC4243043

[134] Christensen C.L., Kwiatkowski N., Abraham B.J., Carretero J., Al-Shahrour F., Zhang T., Chipumuro E., Herter-Sprie G.S., Akbay E.A., Altabef A. et al. Targeting transcriptional addictions in small cell lung cancer with a covalent CDK7 inhibitor. Cancer Cell. 2014; 26:909–922. 25490451 10.1016/j.ccell.2014.10.019 PMC4261156

[135] Pott S., Lieb J.D. What are super-enhancers. Nat. Genet. 2015; 47:8–12. 25547603 10.1038/ng.3167

[136] Zamudio A.V., Dall’Agnese A., Henninger J.E., Manteiga J.C., Afeyan L.K., Hannett N.M., Coffey E.L., Li C.H., Oksuz O., Sabari B.R. et al. Mediator condensates localize signaling factors to key cell identity genes. Mol. Cell. 2019; doi:10.1016/j.molcel.2019.08.016. 10.1016/j.molcel.2019.08.016 PMC6898777 31563432

[137] Shin H.Y., Willi M., HyunYoo K., Zeng X., Wang C., Metser G., Hennighausen L. Hierarchy within the mammary STAT5-driven Wap super-enhancer. Nat. Genet. 2016; 48:904–911. 27376239 10.1038/ng.3606 PMC4963296

[138] Bahr C., von Paleske L., Uslu V.V., Remeseiro S., Takayama N., Ng S.W., Murison A., Langenfeld K., Petretich M., Scognamiglio R. et al. A Myc enhancer cluster regulates normal and leukaemic haematopoietic stem cell hierarchies. Nature. 2018; 553:515–520. 29342133 10.1038/nature25193

[139] Shi J., Whyte W.A., Zepeda-Mendoza C.J., Milazzo J.P., Shen C., Roe J.S., Minder J.L., Mercan F., Wang E., Eckersley-Maslin M.A. et al. Role of SWI/SNF in acute leukemia maintenance and enhancer-mediated Myc regulation. Genes Dev. 2013; 27:2648–2662. 24285714 10.1101/gad.232710.113 PMC3877755

